# Prognostic implications of the Quebec Task Force classification of back-related leg pain: an analysis of longitudinal routine clinical data

**DOI:** 10.1186/1471-2474-14-171

**Published:** 2013-05-24

**Authors:** Alice Kongsted, Peter Kent, Tue Secher Jensen, Hanne Albert, Claus Manniche

**Affiliations:** 1 Research Department, The Spine Centre of Southern Denmark, Middelfart, Hospital Lillebaelt, Institute of Regional Health Services Research, University of Southern Denmark, Part of Clinical Locomotion Network, Ostre Houghvej 55, Middelfart 5500, Denmark

**Keywords:** Classification, Longitudinal studies, Low back pain, Prognosis, Sciatica

## Abstract

**Background:**

Low back pain (LBP) patients with related leg pain have a more severe profile than those with local LBP and a worse prognosis. Pain location above or below the knee and the presence of neurological signs differentiate patients with different profiles, but knowledge about the prognostic value of these subgroups is sparse. The objectives of this study were (1) to investigate whether subgroups consisting of patients with *Local LBP only*, *LBP + leg pain above the knee*, *LBP + leg pain below the knee*, and *LBP + leg pain and neurological signs* had different prognoses, and (2) to determine if this was explained by measured baseline factors.

**Methods:**

Routine clinical data were collected during the first visit to an outpatient department and follow-ups were performed after 3 and 12 months. Patients were divided into the four subgroups and associations between subgroups and the outcomes of activity limitation, global perceived effect (GPE) after 3 months, and sick leave after 3 months were tested by means of generalised estimating equations. Models were univariate (I), adjusted for duration (II), and adjusted for all baseline differences (III).

**Results:**

A total of 1,752 patients were included, with a 76% 3-month and 70% 12-month follow-up. Subgroups were associated with activity limitation in all models (p < 0.001). *Local LBP* had the least and *LBP + neurological signs* the most severe limitations at all time-points, although patients with neurological signs improved the most. Associations with GPE after 3 months were only significant in Model I. Subgroups were associated with sick leave after 3 months in model I and II, with sick leave being most frequent in the subgroup with neurological signs. No significant differences were found in any pairwise comparisons of patients with leg pain above or below the knee.

**Conclusions:**

Subgrouping LBP patients, based on pain location and neurological signs, was associated with activity limitation and sick leave, but not with GPE. The presence of neurological signs and pain in the leg both have prognostic implications but whether that leg pain without neurological signs is above or below the knee does not.

## Background

It is widely believed that the identification of homogeneous subgroups of low back pain (LBP) patients is important for the optimal prediction of prognosis and care [1,2]. LBP patients with leg pain are reported to differ from those with local LBP only, both in terms of general clinical characteristics and prognosis. However, patients with leg pain do not form a single homogenous group and the association between leg pain and prognosis is quite weak [3-5]. More detailed examination of subgroups within the leg pain population [6,7] may lead to identification of stronger prognostic indicators.

LBP-related leg pain has been subgrouped into pain above the knee, pain below the knee and leg pain with neurological signs [6]. Classifying patients into these three subgroups and patients with local LBP only, as described by the Quebec Task Force on spinal pain (QTF), has shown associated differences on a number of clinical characteristics that display a generally increasing severity from patients with local LBP, across the categories of leg pain above the knee and below the knee, to patients with neurological signs [8-10]. These subgroups have also been demonstrated to differ on the outcomes of pain and activity limitation in one study involving a clinical population [11], and on pain, activity limitation and work loss in a study conducted in a workplace setting [12]. These studies did not investigate the extent to which the predictive value of subgrouping was explained by differences in baseline characteristics. In two studies from primary care comparing outcomes on physical and psychological symptoms between the subgroups of local LBP, LBP with leg pain above the knee, and LBP with leg pain below the knee, patients with leg pain below the knee had the worst outcomes [13,14]. Other baseline factors accounted for most of the differences observed between subgroups in one of these studies [13], but not in the other [14].

Differentiation between leg pain above and below the knee and leg pain with and without neurological signs were suggested as diagnostic tools more than twenty years ago [6], but still there is sparse knowledge about their clinical relevance for prognosis or as treatment effect moderators. A recent systematic review of the impact of LBP-related leg pain on outcomes concluded that leg pain appears to be associated with worse outcomes, but highlighted the lack of evidence concerning leg pain subgroups [15]. Furthermore, the reviewed studies did not allow the authors to assess whether the presence of leg pain was an independent predictor of outcome.

The objective of the current study was to investigate whether the four QTF subgroups were associated with activity limitation after 3 and 12 months, global perceived effect after 3 months, or sick leave at 3 months follow-up in patients referred to a secondary care outpatient department, and whether observed associations could be explained by differences in measured baseline characteristics.

## Methods

The setting and baseline data collection have been previously reported in full detail [8]. In short, data collection was part of the daily clinical routine in a secondary care outpatient department seeing approximately 9,000 new spinal pain patients per year. At the first visit to the department, patients completed a questionnaire on a touch screen in the waiting area prior to seeing a clinician. Clinicians entered the results of a core set of clinical variables when examining the patient or following the consultation. Both patient-reported and clinician-reported data were entered directly into the Department’s electronic registry, the SpineData database (Regional Ethics Committee Project ID S-200112000-29). Patients were invited to complete a follow-up questionnaire after 3 and 12 months either electronically or in paper format. Only patients who gave informed consent for their data to be used for scientific purposes were included in the study.

### Study sample

All patients aged 18 years or older who were referred with LBP as their main complaint and were seen in the Department between 10 October 2010 and 30 November 2011 were potential participants. Additional inclusion criteria were that data needed to be available from pain intensity scales, a pain drawing, and from the clinician’s neurological examination in SpineData.

### Baseline characteristics

Baseline factors were chosen from the health domains of pain, activity limitation, psychology and work participation on the basis of their having evidence of a prognostic association with LBP [3,8].

Pain items were: duration (months), previous LBP episodes (yes/no), LBP intensity (averaged 0–10 Numerical Rating Scales (NRS) on present LBP, worst LBP last 14 days and typical LBP last 14 days [16]), intensity of leg pain (measured in the same way as for LBP), and pain irritability (requiring a yes-answer to both ’pain is easily aggravated by physical activity’ and ’it takes a long time before it settles again’ [17,18]).

Activity limitation was measured with the 23-item Roland Morris Disability Questionnaire (RMDQ) [19] and calculated as a proportional score (0% = no activity limitation; 100% = maximum activity limitation) [20].

Work participation was assessed as days on sick leave (days off work during the preceding 3 months due to LBP among patients who had conventional employment - were not unemployed, a student, retired or receiving a pension).

Depressive symptoms were measured by the two PRIME-MD 1000 screening questions [21] using a 0–10 NRS (proportion of patients with a score above 6 on both questions). These cut-points were derived in our setting (unpublished data) based on a comparison with population-based thresholds for the Beck Depression Index [22] and the Major Depression Inventory [23]. Pain-related fear of movement was measured using NRS 0–10 scales (proportion with a total score on two screening questions from the Fear Avoidance Belief Questionnaire equal to or above 14) [24]. This threshold was also derived in an unpublished study in our patient setting based on a comparison with a primary care score threshold (mean plus 1 standard deviation) on the physical activity subscale of the Fear Avoidance Belief Questionnaire.

General health was evaluated using the Euroqol health thermometer (Euroqol VAS) that measures self-reported health status today (0 = worst imaginable; 100 = best imaginable) [25].

### Subgroup classification

The four subgroups were formed using the following definitions.

*Local LBP only:* The pain drawing only included local LBP, and the worst leg pain intensity in the preceding 14 days was zero (0–10 scale).

*LBP + pain above knee:* The pain drawing included pain in the anterior or posterior thigh but no pain in the calf or feet, and the worst leg pain intensity in the last 14 days was one or more (0–10 scale).

*LBP + pain below knee:* The pain drawing indicated pain in the calf and/or foot and the worst leg pain intensity in the last 14 days was at least one.

LBP with signs of nerve root involvement *(LBP + NRI*): The pain drawing included pain below the gluteal folds or the groin, worst leg pain intensity was one or more, and at least one of the following findings was present on the painful side during the clinical examination: muscle weakness, impaired tendon reflexes, altered sensation to touch or pinprick, a straight leg raise test that provoked their familiar leg pain (at 60 degrees or less as judged visually), or a positive prone knee bend test combined with pain to the anterior thigh (Reverse Laségue Test). The term ‘signs of nerve root involvement’ should be considered a label given to patients fulfilling these criteria rather than a definitive diagnostic entity.

If classification was unclear, for example a patient reporting leg pain intensity to be zero but indicating leg pain on the pain drawing, the patient was excluded from the analysis. Such ambiguous reporting was not necessarily due to inaccurate answers since the intensity scale asked about the last 14 days, whereas patients were asked to indicate ‘where your pain is’ in the pain chart.

### Outcome measures

Activity limitation was measured after 3 and 12 months by the RMDQ and converted to a proportional score [20].

Global perceived effect was scored on a 7-point Likert scale (‘much worse’ to ‘much better’) at 3-month follow-up. This was not repeated after 12 months since self-assessment of effects that are based on recall seem to be problematic if asked about longer-term changes [26].

Sick leave was defined as the proportion of those in the working population at baseline (that is, not unemployed, a student, a housewife, retired or receiving a pension) who reported being on sick leave due to LBP at the time of the 3-month follow-up. Data on sick leave were not available at 12 months.

### Data analyses

Baseline characteristics were presented as proportions with 95% confidence intervals (95% CI), means with standard deviations (SD), or medians with interquartile ranges (IQR) depending on the data distribution of the variable.

An association between subgroup and activity limitation was primarily tested in a longitudinal model using population averaged generalised estimating equations (GEE) (family Gaussian, link identity, correlation structure exchangeable) which take into account that measures of activity limitation were repeated. The subgroup variable was introduced as a categorical variable in the analysis with dummy variables that had *local LBP only* as the reference category. In addition, absolute subgroup differences at each follow-up time point were tested for statistical significance in generalized linear models. The associations with global perceived effect and sick leave were tested by means of GEE (family binomial, link function logit). ‘Success’ on global perceived effect being defined as ‘much better’ or ‘better’ and all other categories as ‘failure’.

Associations are presented as unadjusted β-values/Odds ratios with 95% CI (Model I). In addition, estimates adjusted for duration of LBP at the first visit (Model II), and adjusted for duration and factors differing between subgroups at baseline (Model III) were calculated. Only covariates with p < 0.1 were kept in Model III with global perceived effect and sick leave as the outcomes because the number of cases was too low to allow for a larger model. Missing values on baseline variables used as covariates were imputed using multiple imputations by five chained iterations (logit for binary, mlogit for categorical, and predictive mean matching for continuous variables). Seventy-five per cent of patients had no missing data, and most imputed variables had less than 10% imputed data. However, 17% were imputed for pain irritability and 10% for self-reported general health.

Model I was mainly considered relevant to the setting of the study, since differences in duration between subgroups were regarded to be a consequence of referral patterns to the Department rather than related to the condition. Model II answered whether the four QTF subgroups were associated with outcome, and Model III investigated whether an observed association could be explained by differences in measured baseline characteristics. Episode duration was categorised by being split into 25% quartiles since it had a non-linear relationship with the outcome measures.

All analyses were performed using STATA/SE 12.1 using ‘mi’ functions for estimations based on multiple imputations.

## Results

### Study sample

**Figure 1:**
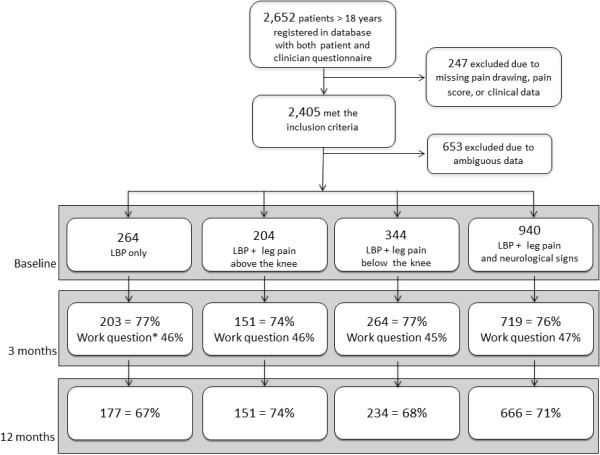
**Flow chart from registration in clinical registry to 12-month follow-up.** * Proportion of working population responding to the sick leave question.

A total of 2,652 LBP patients above 18 years of age were registered in SpineData within the inclusion period of the study (56% females, mean age 50 years). From these 2,405 fulfilled the inclusion criteria and 1,752 (55% females, mean age 50 years) could be classified in the pre-defined subgroups (Figure 1).

Among those with *LBP + NRI*, 669 patients had more than one neurological sign. Of those with only one sign, 73 patients had only a positive straight leg raise, 45 had only reduced muscle strength, 135 had only altered sensation, and 18 had only impaired tendon reflexes.

Follow-up after 3 and 12 months was completed by 76% and 70% respectively, but response rates relating to sick leave were lower (Figure 1). Non-responders did not differ significantly from responders on LBP intensity, duration, activity limitation, depression or fear of movement at baseline. However, non-responders at 3-months follow-up were more often male (48% vs. 43%) and were on average 1.4 years younger as compared with the responders (p < .05). These differences between responders and non-responders did not differ significantly between subgroups. Non-responders at 12-month follow-up were on average 3.3 years younger (p < .05), and did not differ significantly from responders on other baseline factors. Also, non-response to the sick leave question did not differ across subgroups.

### Baseline characteristics

Patient self-reported characteristics are summarised in Table 1. Statistically significant differences across subgroups were observed for all measured baseline factors except fear of movement. Generally, those with *Local LBP* were the least severely affected and those with *LBP + NRI* had the most severe profile. The differences observed in duration indicated that patients with *LBP + NRI* were referred to the Department earlier than other patients, but even in that subgroup, many patients reported very long-lasting pain.

**Table 1:** Patient-reported baseline characteristics

	**Local LBP only**	**LBP + pain above knee**	**LBP + pain below knee**	**LBP + signs of nerve root involvement**	**p-values**
					**For test of any differences across all subgroups**
**Females,** (95% CI)	50% (44–56)	60% (54–67)	61% (56–67)	54% (50–57)	0.01
**Age in years,** median (IQR)	47 (36–58)	45 (36–56)	52 (40–66)	50 (38–62)	<0.001
**Duration in months,** median (IQR)	16 (6–57)	14 (6–38)	12 (5–47)	7 (3–26)	<0.001
**Previous episodes,** (95% CI)	69% (64–75)	77% (71–83)	74% (69–79)	79% (76–81)	0.02
**Pain irritability,** (95% CI)	61% (55–68)	73% (66–80)	69% (63–74)	76% (73–80)	<0.001
**LBP intensity,** median (IQR)	5 (4–7)	6 (4–7)	6 (4–7)	6 (5–8)	<0.001
**Leg pain intensity,** median (IQR)	0	4 (3–6)	6 (4–7)	6 (5–8)	<0.001 (*local LBP* not in test)
**Activity limitation,** median (IQR)	48 (26–66)	57 (39–73)	61 (43–74)	70 (57–83)	<0.001
Any sick leave days in last 3 months*, (95% CI)	42% (34–51)	48% (37–59)	46% (37–55)	55% (50–60)	0.04
**Depressive symptoms,** (95% CI)	12% (8–16)	17% (12–22)	13% (10–17)	20% (18–23)	0.002
**Fear of movement,** (95% CI)	21% (16–26)	24% (18–29)	20% (16–24)	26% (23–29)	0.09
**General health,** mean (SD)	56 (24)	53 (22)	50 (23)	50 (23)	<0.001

* among those in conventional employment n = 1,002.

### Associations between subgroups and activity limitation

**Figure 2:**
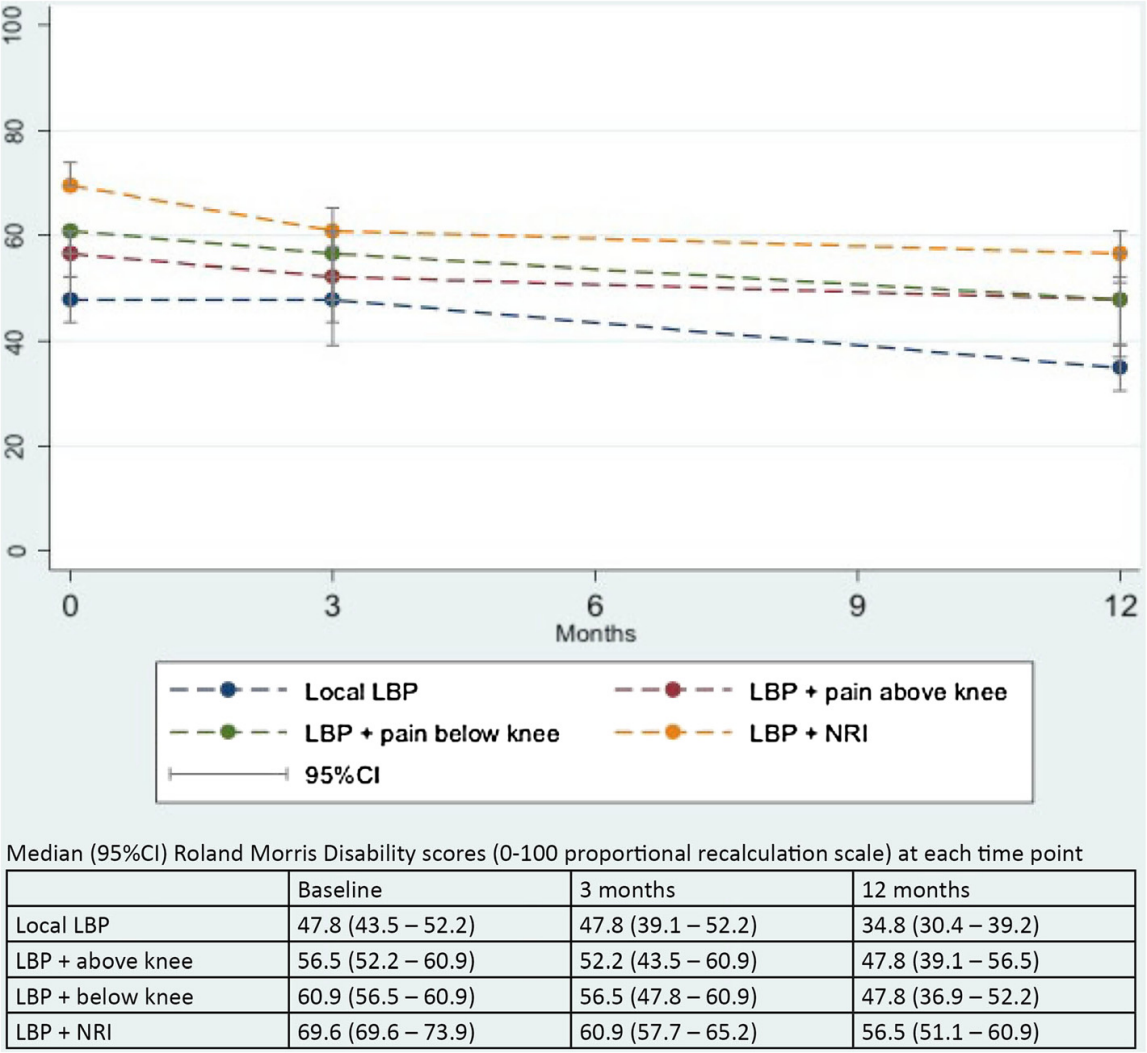
**Mean RMDQ scores in four subgroups at baseline, 3 months, and 12 months.** Activity limitation in four subgroups at the initial visit to the Department and over the clinical course.

Activity limitation from baseline to 12-month follow-up within the four subgroups is illustrated in Figure 2. Statistically significant associations were present between subgroups on change in activity limitation in both the unadjusted and adjusted analyses (Table 2). However, the residual variance was only slightly reduced by subgroups (R^2^ = .04). Patients with *LBP + NRI* improved more than other subgroups in pairwise comparisons, and the estimated effect of being in the *LBP + NRI* subgroup on the course of activity limitation was largely unaltered after adjusting for duration and other covariates.

**Table 2:** Associations between subgroups and activity limitations (Roland Morris Disability Questionnaire 0–100) in longitudinal models (n = 1,745)

	**Beta-coefficients (95% CI)**	**Beta-coefficients (95% CI)**	**Beta-coefficients (95% CI)**
	**Model I: Unadjusted association**	**Model II: Adjusted for duration**	**Model III: Adjusted for duration, age, gender, previous episodes, pain irritability, LBP intensity, leg pain intensity, depression, general health**
**Subgroup effect (at baseline)**			
Local LBP [reference]			
LBP + pain above knee	7.5 (2.8 – 12.2)*	8.0 (3.4 – 12.7)*	2.2 (−2.4 – 6.7)
LBP + pain below knee	9.3 (5.2 – 13.5)*	9.7 (5.6 – 13.8)*	3.4 (−0.9 – 7.8)
LBP + NRI	19.8 (16.2 – 23.3)*^¤ §^	21.0 (17.5 – 24.5)*^¤§^	11.3 (7.1 – 15.4)*^¤§^
**Subgroup X time (at 3 months)**			
Local LBP [reference]			
LBP + pain above knee	−0.2 (−5.1 – 4.7)	−0.2 (−5.1 – 4.7)	0.0 (−4.9 – 4.9)
LBP + pain below knee	−0.3 (−4.6 – 4.0)	−0.4 (−4.6 – 3.9)	−0.2 (−4.5 – 4.1)
LBP + NRI	−6.7 (−10.4 – -3.1)*^¤ §^	−6.4 (−10.0 – -2.4)*^¤§^	−6.3 (−10.0 – -2.7)*^¤§^
**Subgroup X time (at 12 months)**			
Local LBP [reference]			
LBP + pain above knee	2.1 (−2.9 – 7.1)	2.2 (−2.8 – 7.1)	2.2 (−2.7 – 7.2)
LBP + pain below knee	−1.0 (−5.4 – 3.5)	−0.9 (−5.3 – 3.5)	−1.0 (−5.4 – 3.4)
LBP + NRI	−6.3 (−10.1 – -2.5)*^¤§^	−6.2 (−10.0 – -2.4)*^¤§^	−6.3 (−10.0 – -2.5)*^¤§^

Subgroup effect and Subgroup X Time effects: p < 0.001 in all Models I-III; *p < 0.05 in comparison with *Local LBP;*^¤^ p < 0.05 in comparison with *LBP + pain above knee;*^§^p < 0.05 in comparison with *LBP + pain below knee*.

Looking at absolute RMDQ scores, the subgroup with *Local LBP* had the least activity limitation at all time points and the *LBP + NRI* the most (Figure 2). Pairwise comparisons of absolute RMDQ scores adjusted for duration (Model II) were all significant (p < 0.05) except that *LBP + pain above knee* and *LBP + pain below knee* did not differ significantly at any time point. In Model III, *LBP + NRI* differed significantly from *Local LBP* at 3-month follow-up*.* At 12-month follow-up, significant differences existed between *Local LBP* and *LBP + pain above knee* and between *Local LBP and LBP + NRI*. However a very small proportion of the variance in activity limitation was explained by subgroups (R^2^ = .02 for 3- and 12-month analyses).

### Associations between subgroups and global perceived effect

At the 3-month follow-up, 31% of the cohort reported to be ‘much better’ or ‘better’. This proportion varied across the subgroups from 23% in the *LBP + pain below knee* subgroup to 36% in the *LBP + NRI* subgroup. There were statistically significant associations between the subgroups and global perceived effect in Model I but the prognostic capacity in terms of AUC was low, and the association was not significant when duration was taken into account in Model II (Table 3). Therefore Model III was considered irrelevant. The *LBP + NRI* subgroup had higher odds of being ‘much better’ or ‘better’ as compared with the *Local LBP* and the *LBP + pain below knee* subgroups in pairwise comparisons (Table 3).

**Table 3:** Associations between subgroups and general perceived effect after 3 months (n = 1,304)

	**Odds ratios (95% CI)**	**Odds ratios (95% CI)**
	**Model I: Unadjusted association**	**Model II: Adjusted for duration**
**Subgroups**	p 0.001	p = 0.2
Local LBP [reference]		
LBP + pain above knee	1.13 (0.70 – 1.82)	1.06 (0.64 – 1.76)
LBP + pain below knee	0.85 (0.55 – 1.31)	0.76 (0.48 – 1.20)
LBP + NRI	1.58 (1.11 – 2.26)*^§^	1.12 (0.77 – 1.64)
	AUC = 0.57	

Odds ratios for being ‘much better’ or ‘better’ after 3-months.

*p < 0.05 in comparison with *Local LBP.*

^§^p < 0.05 in comparison with *LBP + pain below knee.*

*AUC* Area under the Receiver Operating Characteristic curve.

### Associations between subgroups and sick leave

At baseline 1,003 (57%) of the participating patients were in the working population (*Local LBP only* 64%, *LBP + above knee* 62%, *LBP + below knee* 54%, and *LBP + NRI* 56%. P = 0.03). At the 3-months follow-up 29% (95% CI: 25-33%) of these were currently on sick leave, with the distribution in the subgroups ranging from 19% in the *Local LBP* subgroup to 35% in the *LBP + NRI* subgroup (p = 0.02). A larger proportion of patients in the *LBP + NRI* subgroup were on sick leave at 3 months and subgroups were significantly associated with sick leave in model I and model II but not in model III (Table 4).

**Table 4:** Sick leave among the working population in each subgroup after 3 months (n = 462)

	**Odds ratios (95% CI)**	**Odds ratios (95% CI)**	**Odds ratios (95% CI)**
	**Model I: Unadjusted association**	**Model II: Adjusted for duration**	**Model III: Adjusted for duration, general health, activity limitation**
**Subgroups**	p = 0.02	p = 0.04	p = 0.7
Local LBP [reference]			
LBP + pain above knee	1.44 (0.64 – 3.26)	1.45 (0.64 – 3.28)	1.21 (0.50 – 2.91)
LBP + pain below knee	1.23 (0.57 – 2.63)	1.18 (0.55 – 2.54)	0.84 (0.37 – 1.91)
LBP + NRI	2.25 (1.21 – 4.19)*^§^	2.13 (1.13 – 4.00)*^§^	1.21 (0.61 – 2.43)
	AUC = 0.58	AUC = 0.60	

*p < 0.05 in comparison with *Local LBP.*

^§^p < 0.05 in comparison with *LBP + pain below knee.*

*AUC* Area under the Receiver Operating Characteristic curve*.*

## Discussion

This study investigated whether subgrouping of LBP patients based on leg pain patterns had any prognostic implications. Patients with *LBP + NRI* improved more than other subgroups on change in activity limitation but had a poorer outcome as measured by absolute RMDQ scores after one year. Patients with *Local LBP*, *LBP + pain above knee*, and *LBP + pain below knee* all had similar trajectories of activity limitation. This resulted in similar absolute RMDQ scores for *LBP + pain above knee* and *LBP + pain below knee* patients. In contrast, the *Local LBP* was the subgroup least affected by activity limitation both at baseline and after one year.

There was no significant association between subgroups and global perceived effect above that which could be explained by differences in duration. For the outcome of sick leave, patients in the *LBP + NRI* subgroup had a larger risk of long-lasting sick leave at 3 months compared with patients in the *Local LBP* and *LBP + pain above knee* subgroups.

The larger improvement in activity limitation within the *LBP + NRI* group was not explained by other measured baseline factors. Duration, age, gender, previous LBP episodes, pain irritability, LBP intensity, leg pain intensity, depression, and general health were all taken into account but differences on these factors between subgroups were not shown to be the reason for the different trajectories. Thus, the presence of neurological signs was associated with larger improvement, but at the same time a poorer outcome, and this is likely to be a direct effect of nerve root involvement. The finding that patients with neurological signs report better global perceived effect and poorer absolute outcome has been observed in previous studies that used unadjusted analyses. Those studies included patients from surgical departments [11] and a workplace setting [11,12]. In a primary care cohort, which predictably had a shorter LBP duration than our secondary care cohort, Hill et al. [13] found that prognostic differences between subgroups with *local LBP, LBP + pain above knee, and LBP + pain below knee* were explained by other baseline characteristics. It may be that such baseline characteristics are important covariates early in the clinical course but our results highlight that the inclusion of neurological signs is more prognostically important than only distinguishing between pain above and below the knee, and we believe presence of the *LBP + NRI* subgroup is likely to be a central explanation for why our results differ from those of Hill et al.

A strength of the current study is that data were collected prospectively from a near-complete cohort of people in routine care. We believe this strengthens the generalisability of our results to other chronic LBP populations. Furthermore, the sample size was adequate for the conducted analyses, and data were available that made possible analyses of outcomes across different domains of health. Lastly, the response rates of 76% and 70% at the two follow-up time points, that were very similar in all the studied subgroups, we consider to be acceptable for a clinical registry.

The study also had limitations. The most important limitation from our perspective was the definition of nerve root involvement. Classification into the group with *LBP + NRI* required the presence of just one positive finding in the neurological examination, and the reliability of these findings in our clinical department, notwithstanding an ongoing quality assurance program, is unknown. A lack of such knowledge and less stringent procedures for data collection than are possible in clinical trials are inherent limitations of data from large clinical databases that were not collected for a specific research project. Moreover, for unknown reasons, answers to sick leave questions at follow-up were more often incomplete than other outcome measures. However, this did appear to affect subgroup differences.

Overall, this simple QTF classification of LBP displayed an association with the outcome of activity limitation that was above what could be explained by other measured baseline characteristics, and the QTF subgroups were also associated with sick leave after 3 months when only duration was included as a covariate. Subgroup differences were most marked between *Local LBP* and *LBP + NRI* and sometimes these groups also differed from other groups. However, whether leg pain location was above or below the knee was not an important distinction for the outcome measures investigated.

Despite the QTF classification displaying statistically significant associations at a subgroup level, it explained very little of the variance (2%) in the outcome activity limitation at an individual patient level and the predictive ability relating to sick leave was also low when measured by the AUC statistic. It is not uncommon in LBP that prognostic factors show statistically significant associations with outcome at a group level but little predictive value at an individual level [27] and there is no evidence for a single factor that substantially affects LBP prognosis on its own for all individuals. Also, investigating separate prognostic factors is a necessary step to inform more sophisticated modelling of multiple factors that may be more accurate for individuals. Hayden et al. classified prognostic research as a 3-step sequential process [28]. Initially, factors that are associated with outcome are identified, then tested for their independent effect on outcome, and lastly prognostic pathways are investigated by mapping how prognostic factors, mediators and moderators interact and influence outcome. The current study would be classified as a second step investigation and suggests that leg pain and presence of neurological signs should be included in studying prognostic pathways.

However, another potentially important result from prognostic research is the treatment implications of subgroup-targeted treatment. Classification tools such as the STarT Back Tool have shown that appropriate matching of treatment pathways to prognostic subgroups can result in better patient outcomes that are also cost-effective [29]. It may be that the cost-effectiveness of LBP care can be improved by subgroup-focused treatment of patients, even if those groups, such as QTF subgroups, are not completely homogenous. Put another way, useful improvements in outcomes may result at a clinical population level, even if the predictability of outcome in individuals remains limited. The current study was unable to explore this as treatment was not targeted to the QTF subgroups, but the principle of subgroup-targeted treatment is a promising direction for research.

## Conclusion

In summary, the QTF classification was a prognostic factor at a group level but not very accurate at predicting outcome for individual patients. Therefore this classification should be considered as a factor to be included in multi-factor predictive models, though probably without the distinction of pain above and below the knee. Also, it remains to be investigated whether the QTF classification is a potential treatment effect modifier and its role as a predictor and/or treatment effect modifier may qualify it to be included in multi-dimensional subgrouping tools.

## Competing interests

The authors have no financial or non-financial competing interests to declare.

## Authors’ contributions

AK and PK formed the study idea. All authors were involved in the design of the study, interpretation of data, revision of the manuscript, and gave final approval of the manuscript. AK performed the data analysis and wrote the initial draft of the manuscript.

## Pre-publication history

The pre-publication history for this paper can be accessed here:

http://www.biomedcentral.com/1471-2474/14/171/prepub
