# Correction: prognostic implications of the Quebec Task Force classification of back-related leg pain: an analysis of longitudinal routine clinical data

**DOI:** 10.1186/1471-2474-14-236

**Published:** 2013-08-09

**Authors:** Alice Kongsted, Peter Kent, Tue S Jensen, Hanne Albert, Claus Manniche

**Affiliations:** 1 Research Department, The Spine Centre of Southern Denmark, Middelfart, Hospital Lillebaelt, Institute of Regional Health Services Research, University of Southern Denmark, Part of Clinical Locomotion Network, Ostre Houghvej 55, Middelfart 5500, Denmark

## Background for this correction

In the publication entitled ‘Prognostic implications of the Quebec Task Force classification of back-related leg pain: an analysis of longitudinal routine clinical data’ [1] we described the characteristics of low back pain (LBP) patients classified according to the Quebec Classification for spinal pain [2]. The objectives of the study were to investigate whether subgroups consisting of (a) patients with Local LBP only, (b) LBP and leg pain above the knee, (c) LBP and leg pain below the knee, and (d) LBP with leg pain and neurological signs had different prognoses, and to determine if this was explained by measured baseline factors. The included cohort was from a secondary outpatient department. Routine clinical data were collected in an electronic database at the first visit and follow-ups were performed after 3- and 12-months. The study concluded that subgrouping of LBP patients, based on pain location and neurological signs, was associated with activity limitation and sick leave, but not with global perceived effect. Both the presence of neurological signs and having leg pain did have prognostic implications, but whether the reported leg pain was located above or below the knee did not.

In the paper we reported that all patients aged 18 years or older who were referred with LBP as their main complaint and were seen in the Department between 10 October 2010 and 30 November 2011 were potential participants. Other inclusion criteria were that data needed to be available from pain intensity scales, a pain drawing, and from the clinician’s neurological examination in SpineData. However, unfortunately we had overlooked that an additional selection criterion for this cohort was that patients needed to have completed at least one follow-up questionnaire.

### Implications of this reporting error

Selection of only those who completed a follow-up meant that the reported response rates (76% at 3-months and 70% at 12-months) were artificially inflated. Those response rates should be interpreted as: Of those patients who responded to at least one follow-up questionnaire, 76% completed 3-months outcomes and 70% completed the 12-months outcomes. The overall response rates based on the source population were actually 58% at 3-months and 55% at 12-months.

This cohort selection may have introduced a selection bias. However, there were no significant differences in response rates across subgroups at 3-months (57% subgroups (a)–(c), 58% subgroup (d), p = 0.96) or 12-months follow-up (subgroups (a) + (c) 52%, subgroup (b) 59%, subgroup (d) 56%), p = 0.16). Non-responders did not differ significantly from responders on LBP intensity, duration, activity limitation, depression or fear of movement at baseline. However, non-responders at 3-months follow-up were more often male (48% vs. 44%) and were on average 3 years younger as compared to the responders (p < .05). Non-responders at 12-month follow-up were on average 4 years younger (p < .05) than responders, and had slightly more intense LBP (0.2 points) and activity limitation (2 points) at baseline than responders. These differences between responders and non-responders did not differ significantly between subgroups.

In an unpublished study of potential follow-up bias in this clinical population, data were available from 200 consecutive people who had not completed the 12-month follow-up questionnaire but subsequently did so when contacted by a research assistant. Their responses were compared with those of 300 randomly selected patients who did complete the electronic 12-month questionnaire in the same time period. Non-compliers showed no difference in their: (i) baseline age, gender, episode duration, general health, depression, low back pain intensity, activity limitation, sick leave, making a LBP-related insurance claim, physically strenuous work, or job satisfaction, nor their (ii) 12 month activity limitation, change in activity limitation from baseline, or sick leave. However on average, non-compliers had (i) more low back pain intensity at 12-months (5.2 (SD2.6) compared with 4.5 (SD2.7) on a 0–10 scale, p = 0.004), and (ii) had less change in low back pain intensity from baseline to 12 months (0.8 (SD2.5) compared with 1.3 (SD2.7) on a 0–10 scale, p = 0.048). These comparisons were not available for the 3-month data.

## Discussion

This unfortunate reporting error in our previously published paper resulted in response rates that were artificial inflated. The lower actual response rates may have introduced some bias, but from the information available we believe that drop outs did not affect the study conclusions.

## Pre-publication history

The pre-publication history for this paper can be accessed here:

http://www.biomedcentral.com/1471-2474/14/236/prepub
